# Glucocorticoid-Induced Leucine Zipper (GILZ) Regulates Testicular FOXO1 Activity and Spermatogonial Stem Cell (SSC) Function

**DOI:** 10.1371/journal.pone.0059149

**Published:** 2013-03-14

**Authors:** Devi Ngo, Qiang Cheng, Anne E. O′Connor, Kathleen D. DeBoer, Camden Y. Lo, Elaine Beaulieu, Mia De Seram, Robin M. Hobbs, Moira K. O′Bryan, Eric F. Morand

**Affiliations:** 1 Department of Medicine, Centre for Inflammatory Diseases, Monash Medical Centre, Monash University, Clayton, Victoria, Australia; 2 Department of Anatomy and Developmental Biology, Monash University, Clayton, Victoria, Australia; 3 Monash Micro Imaging, Monash University, Clayton, Victoria, Australia; 4 Australian Regenerative Medicine Institute, Monash University, Clayton, Victoria, Australia; Cardiff University, United Kingdom

## Abstract

Spermatogonia stem cell (SSC) self-renewal and differentiation are tightly regulated processes that ensure a continued production of mature sperm throughout male adulthood. In the present study, we investigated the role of glucocorticoid-induced leucine zipper (GILZ) in maintenance of the male germline and spermatogenesis. GILZ was detectable in germ cells of wild type mice on the day of birth, suggesting a role for GILZ in prospermatogonia and SSC pool formation. Gilz KO mice were generated and adult males were azoospermic and sterile. During the first wave of spermatogenesis in Gilz KO mice, spermatogenesis arrested part way through pachytene of meiosis I. Subsequent waves resulted in a progressive depletion of germ cells through apoptosis to ultimately produce a Sertoli cell-only phenotype. Further, in contrast to wild type littermates, PLZF^+^ cells were detected in the peri-luminal region of Gilz KO mice at day 6 post-natal, suggesting a defect in prospermatogonia migration in the absence of GILZ. At age 30 days, transient accumulation of PLZF^+^ cells in a subset of tubules and severely compromised spermatogenesis were observed in Gilz KO mice, consistent with defective SSC differentiation. GILZ deficiency was associated with an increase in FOXO1 transcriptional activity, which leads to activation of a selective set of FOXO1 target genes, including a pro-apoptotic protein, BIM. On the other hand, no evidence of a heightened immune response was observed. Together, these results suggest that GILZ suppresses FOXO1 nuclear translocation, promotes SSC differentiation over self-renewal, and favours germ cell survival through inhibition of BIM-dependent pro-apoptotic signals. These findings provide a mechanism for the effects of GILZ on spermatogenesis and strengthen the case for GILZ being a critical molecule in the regulation of male fertility.

## Introduction

The two main functions of spermatogonial stem cells (SSC) are to maintain the SSC population through self-renewal and to generate differentiating daughter spermatogonia for spermatogenesis [1], [2]. Although the molecular mechanisms underlying the transition of SSCs from self-renewal towards differentiation remain poorly understood, a recent study suggested that Forkhead box O (FOXO) proteins play an important role [3]. FOXO proteins are a family of transcription factors that regulate a wide range of biological processes, from cell proliferation and differentiation [4], to leukocyte recruitment in inflammation [5]. Importantly, Goertz et al reported that either deletion or hyper-activation of FOXO1 can lead to spermatogenic failure, suggesting that very tight control of FOXO1 activity is required to maintain the balance between SSC self-renewal and differentiation.

Glucocorticoid-induced leucine zipper (GILZ) is best known as an anti-inflammatory protein, the expression of which is exquisitely sensitive to glucocorticoids [6]. In recent years, GILZ has attracted significant attention due to the suggestion that it may be able to mimic glucocorticoid inhibition of immune cell activation. Although the majority of GILZ-related research has focused on its anti-inflammatory effects, a role of GILZ in the regulation of cell differentiation [7] and cell death [8] has been reported. GILZ has been found to promote CRM1-dependent FOXO nuclear exclusion in HL-60 cells, and therefore inhibit cell apoptosis [9]. In parallel, the expression of GILZ has been shown to be induced by the FOXO3 member of this family [10]. Given the importance of FOXO proteins in fertility, and a recent report demonstrating GILZ expression during early stages of spermatogenesis [11], we hypothesized that GILZ might impact on fertility via effects on FOXO proteins.

To investigate the effects of GILZ on FOXO proteins, and whether these effects translated into effects on spermatogenesis, we generated a Gilz-knockout (KO) mouse strain. GILZ was expressed in wild-type (WT) mouse germ cells, while male Gilz KO mice were sterile, with a complete absence of mature sperm production, in line with recent independent reports [12], [13], [14]. No evidence of hyper-immune activation was observed, but instead we found that GILZ deficiency promoted a transient accumulation of undifferentiated SSC and had negative effects on the progression of differentiation. Spermatogenesis failed during the pachytene phase of meiosis I, and germ cells were progressively depleted in a process associated with increased FOXO1/BIM-dependent pro-apoptotic signaling, such that adult mice had a Sertoli cell only phenotype analogous to that found in a subset of infertile men. These findings indicate that GILZ contributes to the initiation of SSC differentiation and the completion of spermatogenesis via effects on the FOXO1 pathway. Therefore, this study provides the first in vivo evidence suggesting an inhibitory role of GILZ on FOXO1 activity to regulate the fate of germ cells during SSC formation and differentiation.

## Materials and Methods

### Ethics statement

All animal experiments were performed in accordance with the approval of the Monash University Animal Research Ethics Committee.

### Mouse strains

GILZ-deficient (Gilz-KO) mice were generated on a C57Bl/6 background as described [15]. Briefly, disruption of the Gilz gene in C57BL/6 mouse embryonic stem (ES) cells was achieved via homologous recombination using the LoxP/Cre system [16]. As Gilz is located on the X chromosome, Gilz heterozygous (-/+) females and wild-type (WT) males were bred and male offspring genotyped to select Gilz KO (Gilz^-/Y^) littermates for further studies.

### GILZ expression and localization

Gilz mRNA expression at critical points during post-natal testis development and the establishment of the first wave of spermatogenesis was determined using quantitative PCR (qPCR). Whole testes (post-natal day 6, 10, 14, 30 and 70 days) were homogenized using a PT 1200 CL Polytron homogenizer in RLT buffer (Qiagen, Cologne, Germany) and total mRNA was extracted with the RNeasy mini kit (Qiagen). cDNA was generated using random primers (7.5 µg/µl) and the SuperScript™ III reverse transcriptase (Invitrogen). The gene-specific primer sequences used for qPCR are listed in Table 1. qPCR was performed using Power Sybr^®^ Green PCR Master Mix (Applied Biosystems) with Rotor-Gene 3000 (Corbett Research, Mortlake, NSW, Australia). The level of target gene expression was normalized against 18 s ribosomal RNA (rRNA) expression and results are expressed as the number of mRNA copies per 10^6^ 18 s rRNA copies [17].

**Table 1:** Primer sequences for qPCR.

Genes	Forward (5'-3')	Reverse (5'-3')
18 s	GGATCCATTGGAGGGCAAGT	CGAGCTTTTTAACTGCAGCAACT
Gilz	GGAGGTCCTAAAGGAGCAGATTC	GCGTCTTCAGGAGGGTGTTC
IL1β	GGGCCTCAAAGGAAAGAATC	TACCAGTTGGGGAACTCTGC
TNFα	GCCTCTTCTCATTCCTGCTT	CACTTGGTGGTTTGCTACGA
IL6	TTCCATCCAGTTGCCTTCTT	ATTTCCACGATTTCCCAGAG
Ccl2	CCCCAAGAAGGAATGGGTCC	GGTTGTGGAAAAGGTAGTGG
Ret	TGACTGAGGTGAGAGAACAAGGTT	ACACGTACCATAGCAGCATAAAGG
Lhx1	ACCCAGCTTTCCCGAATCC	GGAGTGAAGGTCACCGTGAGA
Egr4	AGGCACTTCCTTGGGACTGA	CTCGGTACATCCCCAGCTTGT
Sall4	AACAAATGCTGTGCCGAGTTC	CCTCGCTGTCATTCATGATGA
Dppa4	CCAGAACAAATGCTGGAGTTGA	ACTCCTCCCCCGGTTCAGT
Bim	GGGCCCCTACCTCCCTACA	CCGCAGCTCCTGTGCAAT

The localization of GILZ protein within the testis was determined immunohistochemically by diaminobenzidine (DAB) using rabbit anti-GILZ (1∶200, FL-134, Santa Cruz Biotechnology, Santa Cruz, CA) followed by secondary and tertiary antibodies (Vectastain kit) as described previously [18].

### Characterization of fertility

The testicular phenotype in Gilz KO males was defined using the strategy outlined in Borg et al [19]. Briefly, testes and epididymides harvested from male WT and Gilz KO littermates at 6, 14, 20 and 30 days post-natal and adult mice were fixed in Bouin's solution and processed into paraffin. Sections were stained with periodic acid Schiff's reagent (PAS) and assessed with reference to the normal histology observed in WT littermates as described previously [20]. Cells undergoing apoptosis were visualized using the Apoptag kit (Millipore, Billerica, MA) as recommended by the manufacturer.

### Meiotic spreading

Meiotic spreads were performed as described in Reinholdt et al [21] and synaptonemal complex formation visualized using an anti-mouse SYCP3 primary antibody (1∶50) (sc-74569, Santa Cruz) and an Alexa Fluor 488 donkey anti-mouse IgG secondary antibody (1∶800) (A11034, Invitrogen).

### Determination of the integrity of blood testis barrier

The structure of the blood testis barrier was assessed using immunohistochemistry for the ectoplasmic specialization marker espin and by electron microscopy as described previously [22].

### Analysis of anti-germ cell immune response

The presence of systemic immune responses to germ cell antigens was assessed by Western blotting as described in [23]. In addition, the presence of inflammation in the adult testis was investigated by staining sections for the pan-immune cell marker CD45, performed by standard DAB immunohistochemistry using rat anti-mouse CD45 (1∶100) (Ly-5, BD) primary antibody, followed by rabbit anti-rat IgG Biotin secondary antibody and finally swine anti-rabbit IgG HRP (both 1∶100 and from DAKO). Finally, the effect of GILZ deficiency on immune responses in the testis was assessed by measuring gene expression of pro-inflammatory cytokines (IL1β, TNFα and IL6) and the chemokine Ccl2 in testicular tissues.

### Detection of PLZF and SALL4 by immunohistochemistry

Tissue was fixed in 4% paraformaldehyde fixative prior to embedding in paraffin. Sections were processed for PLZF and SALL4 immunohistochemistry as described [24]. Anti-PLZF (clone 9E12 [24]) was used at 1∶800 dilution while anti-SALL4 polyclonal antibody (AbCam; ab29112) was used at 1∶1000 dilution.

### Immunofluorescence microscopy

FOXO1 and GILZ co-localization was performed on paraffin sections of testis collected from day 6 post-natal mice. Antigen retrieval was performed using sodium citrate buffer as previously described [18]. Slides were washed 3 times for 5 minutes with PBS and blocked with 1% BSA/PBS for 2 hours room temperature (RT). Slides were incubated with rabbit anti-FOXO1 (1∶50) (C29H4, Cell Signaling) and an anti-GILZ (1∶200) (G5, SantaCruz) mouse monoclonal antibodies overnight at 4°C, followed by the secondary antibodies Alexa Fluor 488 goat anti-rabbit and Alexa Fluor 568 goat anti-mouse (Both 1∶800 and from Invitrogen) for 1 hour RT. The nucleus was stained with NucBlue Live Cell stain (Invitrogen) for 20 minutes at RT and mounted with fluorescence mounting medium (DAKO). Images were acquired 1024×1024 pixels each 12bit using a Nikon C1 confocal laser scanning microscope on a Ti-E base. A 40×1.0NA objective was used for detection with DAPI (emission filter 450/35 nm), Alexa488 (emission filter 515/30) and Texas Red (emission filter 605/75) excited with 405 nm, 488 nm and 561 nm lasers specifically. FOXO1 positive cells were counted using Imaris v7.4.2 (Bitplane AG). Briefly, the Spot analysis module was used to identify individual FOXO1 positive cells based on object size (8 µm diameter) and FOXO1 staining intensity. FOXO1 positive cells were then categorized into nuclear-localizing and cytoplasm-localizing population by utilising the ‘Intensity Centre’ parameter of the analysis, where nuclear-localising FOXO1 cells have a significantly higher ‘Intensity Centre’ than cytoplasm-localising FOXO1 cells. Cell numbers were then expressed as a percentage of total FOXO1 positive cells to normalize against different cell numbers. In a separated set of experiments, the expression/localization of FOXO3 was also examined with rabbit anti-FOXO3 antibody (1∶50) (75D8, Cell Signaling) using the confocal microscope mentioned above.

### Western blot analysis

Day 6 old WT and Gilz KO testes were homogenized in RIPA lysis buffer (Cell Signaling) using a PT 1200 CL Polytron homogenizer. Immunoblotting was performed using antibodies directed against mouse GILZ (1∶3000) (G5, Santa Cruz), BIM (1∶1000, C34C5) and β-actin (1∶10,000, 8H10D10) (both from Cell Signaling).

## Results

### GILZ is expressed during the early periods of spermatogenesis

**Figure 1:**
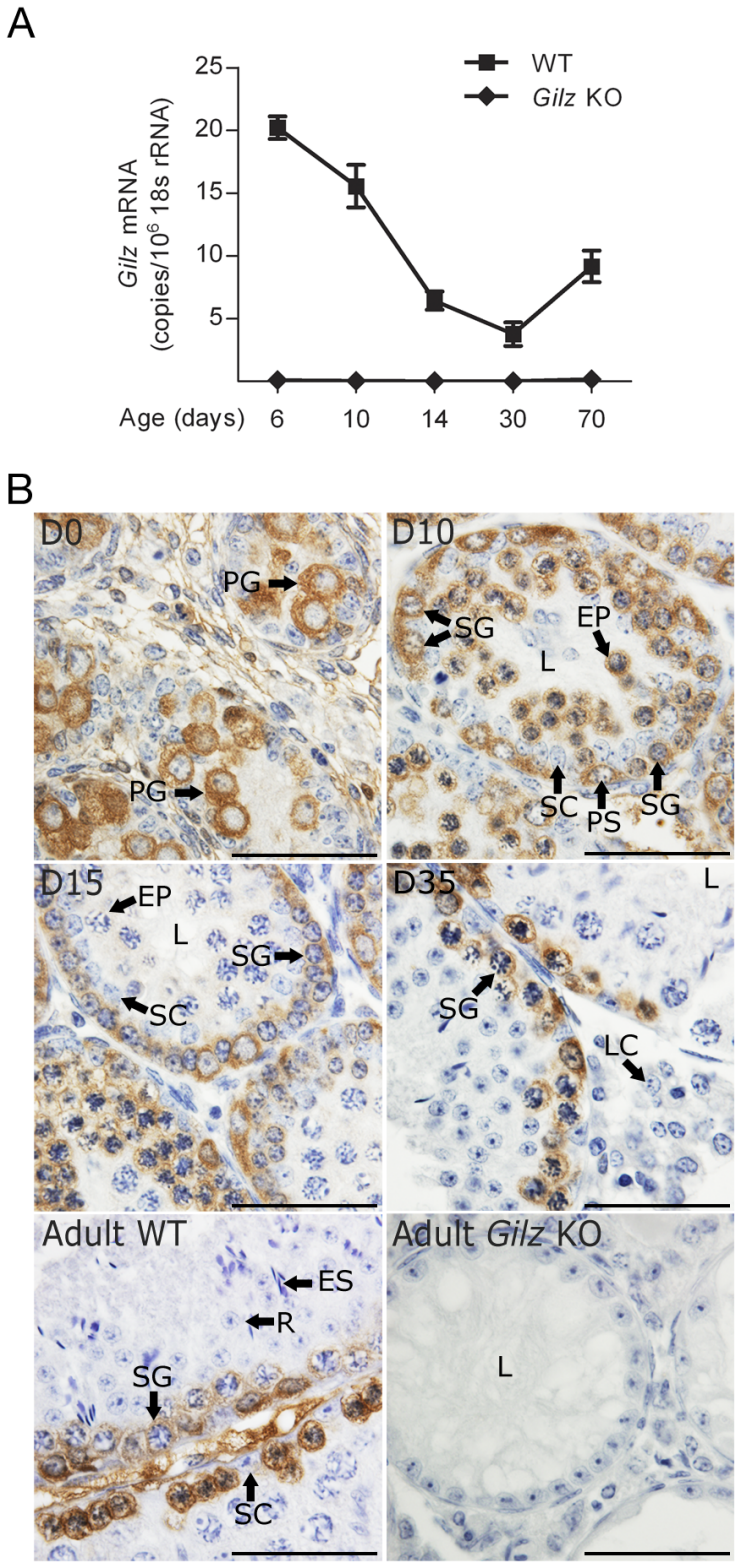
GILZ expression during spermatogenesis. A: Quantitative PCR was undertaken on WT and Gilz KO testes for Gilz mRNA expression at the ages of day 6, 10, 14, 30 and 70. Data represents the mean ± SEM of 3–7 mice per group. B: Immunohistochemistry of WT testes at various ages shows that GILZ protein expression during spermatogenesis. Gilz KO adult testes were also included. Scale bars represent 50 µM. L-lumen, PG-prospermatogonia, SG-spermatogonia, PS-preleptene spermatocytes, R-round spermatids, ES-elongated spermatids, LC-leydig cells, EP-early pachytene, Le-leptotene, SC-Sertoli cells.

Previous microarray studies suggested that Gilz mRNA was expressed at early periods of spermatogenesis [11]. To define in more detail, the expression of GILZ mRNA levels were measured by quantitative PCR (qPCR) on testis samples taken at key points during the establishment of the first spermatogenic cycle in WT mice. As shown in Figure 1A, Gilz mRNA was abundantly expressed at day 6 post-natal, at which point the SSC pool is first formed [25]. After day 6, Gilz expression was reduced and remained so from day 14 onwards. These results are consistent with Gilz being expressed predominantly within early germ cells including spermatogonia.

In order to confirm the above interpretation we next used immunohistochemistry to examine GILZ localization during spermatogenesis in WT mice. Consistent with the mRNA expression, abundant GILZ protein was observed in prospermatogonia on the day of birth (Figure 1B), suggesting the involvement of GILZ in regulation of prospermatogonia function and formation of the SSC pool. Staining of sections from WT mice of increasing age revealed that GILZ was present in spermatogonia, preleptotene through to zygotene spermatocytes, and in early (up to mid-pachytene) but not late pachytene spermatocytes (Figure 1B). There was no observable GILZ protein in haploid germ cells, peritubular cells or Sertoli cells (adult) (Figure 1B). The complete absence of staining in testes from knockout animals confirmed the specificity of the GILZ antibody (Figure 1B).

### GILZ deficiency results in spermatogenic failure

**Figure 2:**
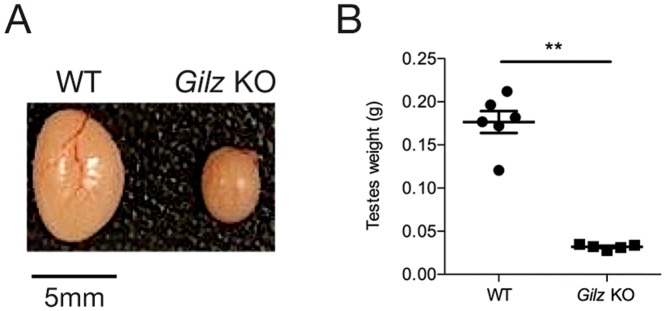
Effect of GILZ deficiency on testicular size. A: Representative image of WT and Gilz KO testes. B: Testes weights of WT and Gilz KO testes. Data represents the mean ± SEM of 5 mice per group. **P<0.001.

**Figure 3:**
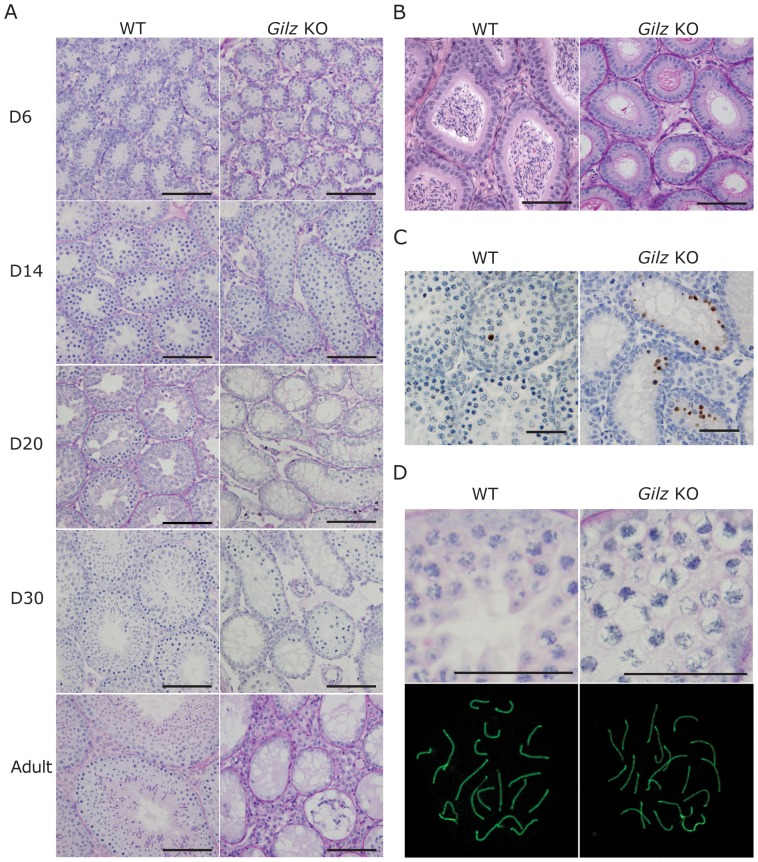
GILZ deficiency leads to spermatogenic failure. A: Testes were collected from 6, 10, 14, 20 and 30 day old and adult WT and Gilz KO mice, processed and stained with PAS. Scale bars represent 100 µM. B: The presence of mature sperm was detected in adult WT but not Gilz KO epididymides. Scale bars represent 100 µM. C: Representative images of TUNEL positive cells in 20 day old WT and Gilz KO testes. Scale bars represent 50 µM. D: Chromatin morphology of pachytene cells was examined using PAS staining (scale bars represent 50 µM) and SCP3 immunofluorescence in day 20 WT and Gilz KO testes.

To further define the role of GILZ in SSC function, we generated Gilz KO mice. While Gilz^-/X^ female mice had normal fertility, Gilz KO male mice were sterile upon mating with WT females. Adult Gilz KO testes were observed to be markedly smaller than those of WT mice (Figure 2A), reflected in a significant reduction in testis mass compared to WT testes (Figure 2B). Histological examination revealed that adult (day 70 post-natal) male Gilz KO testes contained a seminiferous epithelium completely devoid of germ cells, i.e. a Sertoli cell only phenotype (Figure 3A). Of note, these tubules contained a lumen and Sertoli cells with a lacy vacuolated appearance, suggesting that they were of an adult phenotype and may once have harboured germ cells. In order to explore this possibility, testes were collected from mice aged 10, 14, 20 and 30 days. In WT mice, germ cells appeared with the predicted timing (Figure 3A). By contrast, in Gilz KO male testis spermatogenesis was initiated but failed part way through pachytene of meiosis I (Figure 3A). The epithelium in 14 day old mice appeared relatively normal in comparison to WT testis (Figure 3A), however, by 20 days post-natal ∼40% of tubules were Sertoli cell only (Figure 3A). Haploid germ cells were never observed in any GILZ-deficient mice. As anticipated, epididymides from WT animals contained large numbers of sperm, whereas Gilz KO epididymides contained no germ cells (Figure 3B). These data suggest that loss of GILZ resulted in a block during meiosis, in addition to a progressive degeneration of the seminiferous epithelium. The later is suggestive of a progressive failure in SSC or spermatogonial maturation and survival.

### GILZ deficiency is associated with increased apoptosis during spermatogenesis

To determine whether the depletion of germ cells in Gilz KO testis subsequent to meiosis failure was caused by apoptosis, we next examined the presence of apoptotic cells using TUNEL staining. At day 20 post-natal, GILZ deficiency markedly increased TUNEL-positive cells in the testis (Figure 3C), suggesting that non-surviving cells in Gilz KO testis died via apoptosis. Quantitative analysis showed that the number of TUNEL-positive cells was significantly (*p*<0.05) higher in Gilz KO testes comparing to WT controls (data not shown). In addition, apoptotic germ cells were also assessed at day 6 and 14 post-natal. Comparing to WT controls, a similar increase of TUNEL-positive cells in *Gilz* KO testes to that observed at day 20 was detected at day 14, whereas no difference was found at day 6 post-natal (data not shown).

### Disruption of meiosis in Gilz KO testes

Meiotic cells within Gilz KO mice were overtly abnormal. Cells appeared swollen and contained spaghetti-like chromatin (Figure 3D). In order to examine chromosome dynamics of prophase I of meiosis, meiotic spreads were prepared and stained for the synaptonemal marker SCP3. SCP3 is a component of the synaptonemal complex that forms between homologous chromosomes. Within wild type cells the synaptonemal complex is fully formed by the pachytene phase of prophase I. By the diplotene phase the distal ends are beginning to de-synapse [26]. Spermatocytes from Gilz KO males appeared to pair and synapse normally (Figure 3D). Consistent with the histological analysis, diplotene spermatocytes were rarely observed.

### Lack of evidence of anti-germ cell immune response in Gilz KO testes

**Figure 4:**
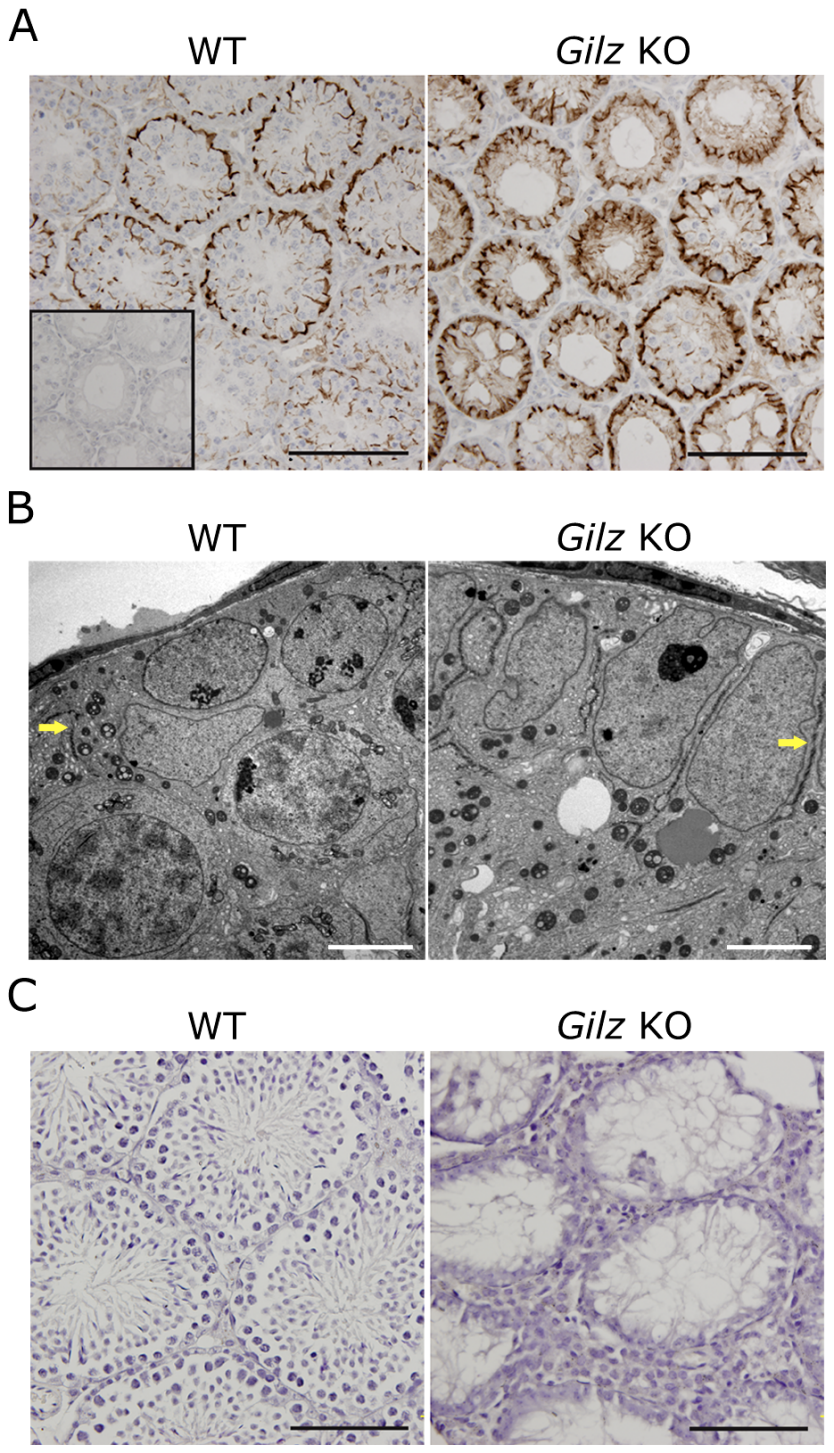
Effects of GILZ deficiency on blood-testis barrier and testicular leukocyte. A: Immunostaining for the blood-testis barrier marker espin was performed on day 20 WT and Gilz KO testes using anti-espin antibody. Scale bars represent 100 µM. Single insert represents the no espin staining negative control. B: Blood-testis-barrier (indicated by arrows) in day 20 old WT and Gilz KO testes samples were examined using transmission electron microscopy. Scale bars represent 5 µM. C: The presence of leukocytes in adult (day 70) WT and Gilz KO testes was examined by immunohistochemistry using CD45 as a marker. Scale bars represent 100 µM.

Given the association between GILZ and immune regulation [6], we hypothesized that GILZ deficiency could be associated with a breakdown of the blood-testis barrier, and consequently an immune response against germ cell antigens normally sequestered behind this barrier [27]. As such, we examined the blood-testis barrier in WT and Gilz KO mice using the marker protein espin and electron microscopy. Immunohistochemical labeling for espin suggested that the blood-testis barrier was positioned in the appropriate location and appeared at the appropriate time in Gilz KO testis (Figure 4A). Of note, because of the absence of haploid germ cells in Gilz KO males, espin staining towards the lumen, wherein the apical ectoplasmic specialization is located, was not observed in Gilz KO animals. The normal structure of the blood-testis-barrier was further confirmed by electron microscopy (Figure 4B, indicated by arrows) wherein no difference between WT and Gilz KO testis was observed.

To further investigate the possibility of a heightened immune response to germ cells in Gilz KO mice, we investigated the presence in the serum of anti-germ cell antibodies and in the testis of inflammatory cells. Serum from both WT and Gilz KO males contained no detectable anti-germ cell antibodies (data not shown). Similarly, no leukocytes (CD45^+^ cells) were detected within the seminiferous epithelium of either WT or Gilz KO animals (Figure 4C). A mouse spleen section was included as a positive control (data not shown). Consistent with this, when compared to WT controls, no significant increase of gene expression of pro-inflammatory cytokines *IL-1β*, *TNFα*, *IL6* or the chemokine *Ccl2* was observed in Gilz KO testes (Supplemental Figure 1). Therefore, these data indicate that GILZ is not a major regulator of testicular immune privilege and that the infertility observed in Gilz KO males was not due to abnormal immune response. Of note, these results are consistent with the recent observation that despite effects on T cell IFN-γ and IL17 production, GILZ deficiency is not associated with increased inflammatory responses to innate or adaptive immune stimulation in vivo [15].

### Presence of SSC in Gilz KO testis

**Figure 5:**
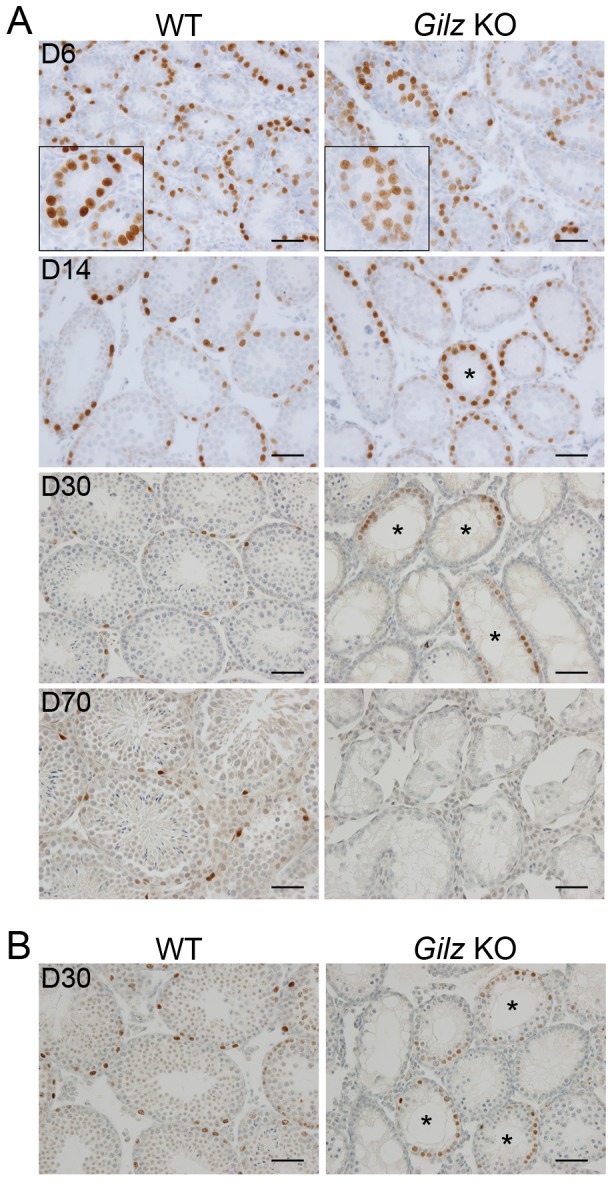
Analysis of the SSC population in Gilz KO testis. A: Immunohistochemistry for PLZF on testis sections from mice of the indicated postnatal ages (days). Higher magnification insets show the presence of PLZF^+^ cells in the periluminal region of day 6 tubules. B: Immunohistochemistry for SALL4 on testis sections from post-natal day 30 mice. Asterisks in A and B indicate day 14 and 30 tubules containing PLZF and SALL4-positive cells respectively. Scale bars represent 50 µM.

The progressive degeneration of the seminiferous epithelium in Gilz KO testes was suggestive of a possible defect in SSC formation and/or the initiation of spermatogenesis. To test this hypothesis, we examined the presence of undifferentiated spermatogonia / SSCs in WT and Gilz KO tubules using PLZF and SALL4 as SSC markers [24]. By day 6 post-natal, SSCs were located on the basement membrane in WT tubules (Figure 5A). However, in Gilz KO tubules, PLZF^+^ cells were still detected in the lumen (Figure 5A), suggesting that GILZ deficiency disrupted or delayed prospermatogonia migration towards the basement membrane and the subsequent development of SSCs. By 14 days post-natal, accumulated PLZF^+^ cells were clearly seen in a subset of Gilz KO tubules (Figure 5A), suggesting that GILZ normally suppresses SSC proliferation or is required for SSC differentiation. Similarly, accumulated PLZF^+^ cells were detected in day 30 old Gilz KO tubules, although most of the tubules were lacking germ cells. It was also noted that, compared to WT, multilayer spermatogenic differentiation was substantially reduced in Gilz KO tubules containing PLZF^+^ cells, suggesting a role of GILZ in regulation of the balance between SSC self-renewal and differentiation. Notably, day 30 Gilz KO tubules were also found to contain cells expressing SALL4, a transcription factor expressed by SSCs [24], providing further evidence for the presence of undifferentiated spermatogonia (Figure 5B). By day 70 post-natal, no PLZF^+^ cells were detected in GILZ deficient tubules (Figure 5A), suggesting that long-term maintenance of SSC survival is GILZ-dependent.

### GILZ deficiency is associated with increased FOXO1 nuclear translocation and transcriptional activity

**Figure 6:**
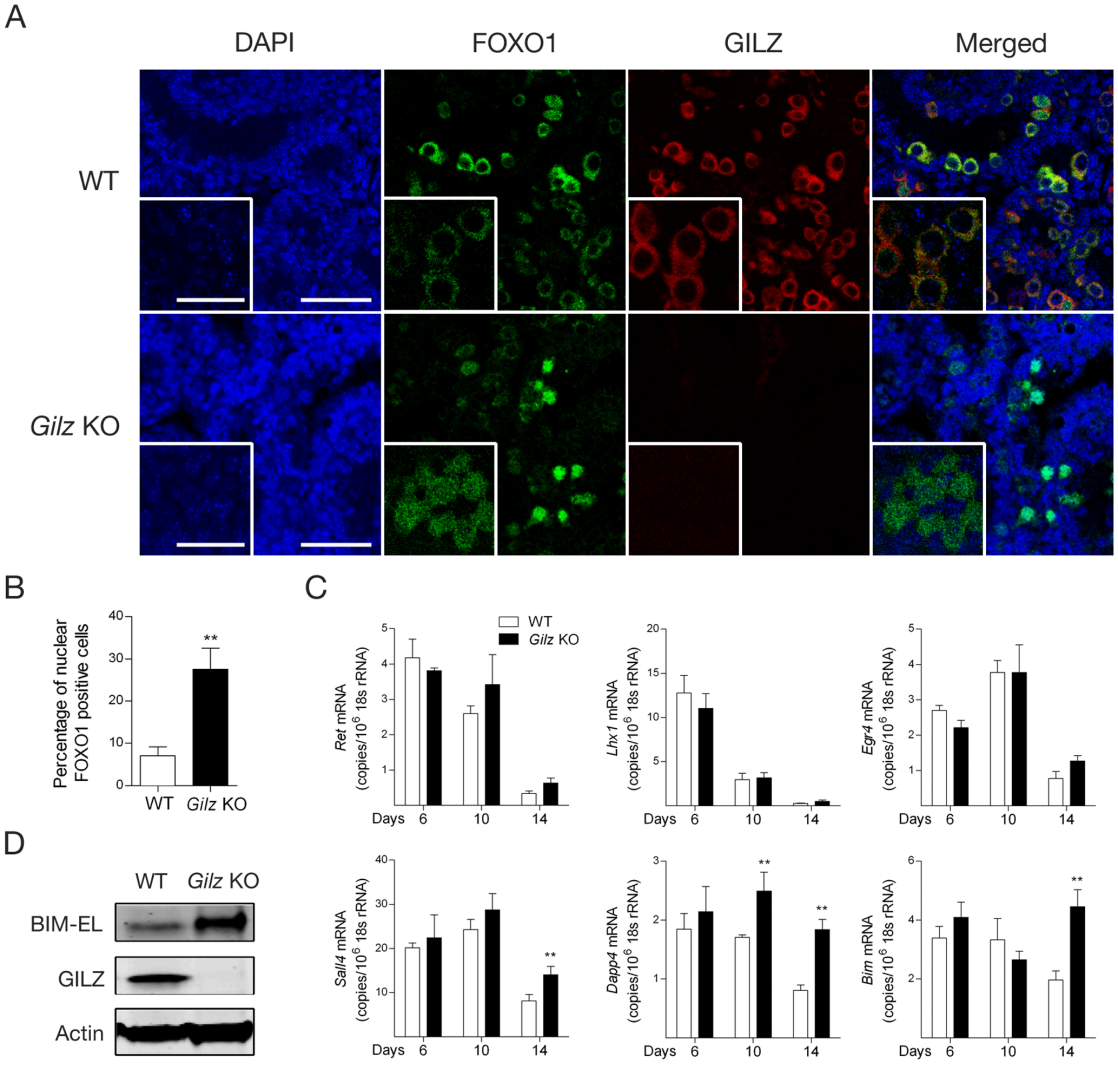
Effect of GILZ deficiency on FOXO1 activity during spermatogenesis. A: Day 6 old WT and Gilz KO mouse testes were used in immunofluorescence analysis to assess the localization of FOXO1 (green) and GILZ (red) using confocal microscopy (DAPI-stain nuclei shown in blue). High magnification inserts showing localization of FOXO1 and GILZ were also included. Scale bars represent 100 µM and insert scale bars represent 25 µM. B: Quantitative analysis of nuclear FOXO1 positive cells in day 6 old WT and Gilz KO testes. Data represents the mean ± SEM of 6 testes. **, p<0.01. C: Quantitative PCR analysis of *Ret*, *Lhx*, *Egr4*, Sall4, Dppa4 and Bim mRNA expression in day 6, 10 and 14 old WT and Gilz KO testes. Results are expressed as the number of mRNA copies per 10^6^ 18 s rRNA copies. Data represents the mean ± SEM of 4–8 mice per group. **, p<0.01 related to WT controls. D: Protein expression of BIM-EL (extra long) and GILZ was detected in day 20 old WT and Gilz KO testes using Western blotting. Representative images of two individual experiments.

FOXO proteins are transcription factors that play an important role in the regulation of SSC renewal and differentiation [3], and it has been reported that GILZ can both positively and negatively affect FOXO protein expression and function [8], [9]. Given GILZ is highly expressed during SSC formation, we next examined whether FOXO1 activity during SSC formation and differentiation was affected by GILZ deficiency. In WT tubules (day 6 post-natal), FOXO1 was detected mainly in the cytoplasm (Figure 6A), suggesting FOXO1 was mainly in its inactivated form. FOXO1 was co-expressed with GILZ (Figure 6A), suggesting GILZ is expressed in undifferentiated SSCs [3]. Although comparable overall expression of FOXO1 was detected in WT and Gilz KO seminiferous tubules, increased nuclear localization of FOXO1 was observed in Gilz KO testis (Figure 6A), suggesting GILZ normally inhibits FOXO1 nuclear translocation. Quantitative analysis showed that nuclear FOXO1 was detected in <10% of WT cells but was significantly increased, to almost 30%, in Gilz KO cells (p<0.01) (Figure 6B). Of note, consistent with observation from another group [3], no expression of FOXO3 and FOXO4 was detectable in either WT or *Gilz* KO testis (data not shown), suggesting FOXO1 is the predominant FOXO protein within the testis.

We next examined the effect of GILZ deficiency on the mRNA expression of Ret, Lhx, Egr4, Sall4 and Dppa4, genes whose expression is significantly reduced in FOXO1-deficient testis [3], and on Bim, a direct FOXO1 transcriptional target [28]. As shown in figure 6C, the expression of Sall4, Dppa4 and *Bim* was significantly higher in Gilz KO testis compared to WT controls at day 14 post-natal, and Gilz KO testis also showed higher expression of *Dppa4* at day 10 post-natal. On the other hand, we found no difference in expression of Ret, Lhx and Egr4 between WT and Gilz KO testes (Figure 6C). These results indicate that FOXO1 nuclear translocation associated with GILZ deficiency led to a selective activation of FOXO1 target genes. Given that BIM is a pro-apoptotic protein, we next examined whether BIM protein expression was increased in Gilz KO testes. As shown in figure 6D, BIM protein expression was markedly up-regulated in GILZ-deficient testes. These findings indicate that in normal testis GILZ suppresses FOXO1-dependent expression of genes including BIM, contributing to the regulation of spermatogonia survival.

## Discussion

GILZ was first identified as a glucocorticoid-induced protein, and its functions as a transducer of anti-inflammatory signals have been reported in a wide range of cells during the past 15 years. GILZ is known to interact with, and inhibit the function of, the Raf and Ras kinases and the NF-κB and AP-1 transcription factors, resulting in inhibitory effects at multiple levels of the cellular response to inflammatory stress [6]. In addition, effects of GILZ on cell cycle, differentiation and apoptosis have recently been demonstrated [7], [8], and some of these effects may depend on GILZ promotion of CRM1-dependent FOXO nuclear exclusion [9]. It has been previously demonstrated that glucocorticoid-induced expression of GILZ is amplified by the binding of FOXO3 to the Gilz promoter [10], suggesting a bi-directional interaction between FOXO proteins and GILZ. A recent study demonstrated that FOXO1 regulates the balance between SSC self-renewal and differentiation and that altered expression of FOXO1 thereby results in spermatogenic failure [3]. We therefore formed the hypothesis that loss of GILZ expression would have detrimental effects on male fertility due to resulting aberrant FOXO1 activity. To address this, we generated GILZ-deficient mice to examine the effects of GILZ on FOXO1 activity and spermatogenesis.

The generation of Gilz KO mice led to the observation that male Gilz KO mice are sterile. Reduced testicular size and weight, and a complete absence of mature germ cells, are consistent with recent observations independently reported by other groups that have generated Gilz KO mice [12], [13], [14]. Our findings indicate that although successfully initiated, the progression of spermatogenesis ceased at the pachytene phase of meiosis I. Examination of PLZF expression suggested that SSC formation did occur in the absence of GILZ, but the persistence of PLZF^+^ cells in the tubular lumen of day 6 post-natal GILZ-deficient mice suggested a defect or delay in prospermatogonia migration. This defect was followed by disordered meiosis and the deletion of disordered germ cells by apoptosis.

Given the reported effects of FOXO proteins on fertility, these findings are consistent with the possibility that GILZ effects on spermatogenesis are mediated via effects on FOXO proteins. We report here that FOXO1 nuclear translocation was increased in GILZ-deficient testes, accompanied by increased expression of the FOXO1-dependent genes Sall4, Dapp4 and Bim. These data suggest GILZ deficiency promotes FOXO1 nuclear translocation and thus the expression of FOXO1-dependent genes. Hyper-activation of FOXO1, achieved by knocking out 3-phosphoinositide-dependent protein kinase 1 (Pdk1), was recently reported to promote SSC proliferation and suppress differentiation, leading to spermatogenic failure [3]. In response to FOXO1 hyper-activation, although SSC proliferation was retained, SSC were unable to differentiate into spermatogonia and initiate spermatogenesis. This is strikingly similar to the phenotype we observed here in GILZ-deficient testis, in that accumulating PLZF^+^ SSC were detected in Gilz KO testes but multilayer spermatogenic differentiation was reduced.

In contrast to the findings in Pdk1-deficient mice, multilayer spermatogenic differentiation was observed in some tubules, suggesting GILZ deficiency does not completely mimic the Pdk1-deficient phenotype, which leads to complete arrest of SSC differentiation. The mechanism of the effects of GILZ deficiency on meiosis failure and spermatogonia survival are not as yet known, but increased FOXO1-dependent expression of BIM would be expected to have pro-apoptotic effects. This notion is further supported by the observation that GILZ deficiency led to an increase of apoptotic germ cells at day 14 and 20 post-natal. The delay prior to the observation of increased apoptosis in Gilz KO testis suggests the alternative possibility that the apoptosis observed is appropriate programmed cell death of disordered cells, rather than an effect of GILZ on apoptosis *per se*. It was also noted that the GILZ deficiency-induced FOXO1 transcriptional activity affected some but not all target genes, i.e. Sall4, Dapp4 and Bim but not Ret, Lhx1and Egr4. Indeed, it is known that, after nuclear translocation, nuclear FOXO1 transcriptional activity is regulated by additional protein modification such as acetylation and interaction with other transcription factors [4], [29]. Therefore, further investigation is required to determine the mechanisms by which GILZ deficiency results in selective modulation of FOXO1 transcriptional activity.

In conclusion, we provide evidence that GILZ suppresses FOXO1 nuclear translocation, affects prospermatogonia migration during SSC pool formation, regulates the divergence between SSC self-renewal and differentiation, and contributes to germ cell survival. This provides a novel mechanism for the profound effect of GILZ on male fertility, and may suggest GILZ as a new therapeutic target in human male infertility.

## Supporting Information

Figure S1 **Effects of GILZ deficiency on mRNA expression of pro-inflammatory cytokines and chemokine.** The mRNA expression of *IL1β* (**A**), *TNFα* (**B**) and *IL6* (**C**)and *CCL2* (**D**) was measured by qPCR in day 6, 10 and 14 day old WT and *Gilz* KO testis. *, *p*<0.05 related to WT controls.
(TIF)
